# Supplementary material for: Examining the psychometric properties of the Controlling Coach Behaviors Scale in Chinese Elite Athletes
Source: PLoS One. 2022 Nov 28;17(11):e0277985. doi: 10.1371/journal.pone.0277985 (PMC9704685; doi:10.1371/journal.pone.0277985)
Supplement: Attachment — Submitted filename: Response to Reviewers.docx [file pone.0277985.s001.docx]

**Response to Reviewers**

We sincerely thank the editor and the reviewer for their comments and advice, which we have used to improve the manuscript. Below is a point-by-point response to each comment. The line numbers refer to the revised manuscript.

**Editor**

***Comment 1****:* *I found one expert reviewer to comment on your work. I also read the manuscript myself. The referee considers your work important overall and suggested some points to consider before publication, mostly statistical issues. Therefore, I would invite you preparing a revision of your work that addresses all concerns together with a cover letter that provides point-by-point replies.*

**Response:** We have addressed the reviewer’s concerns about our statistical analyses and the interpretation of the effects. We have incorporated the editor’s and reviewer’s constructive suggestions into the revised manuscript, and we believe we have answered the other questions raised by the reviewers.

**Reviewer**

***Comment 1***: the theoretical classification of coaching styles might be described more clearly. If possible, a table might be introduced where the styles are listed and described in more detail. To name an example what I mean., see the arrangement in this work: doi: 10.3389/fpsyg.2022.867978, see Table 1 and 2.

**Response:** Thank you very much for your support for our manuscript. We have added a table, called Table 1 in the revised manuscript, that summarises coaching styles (p. 3).

***Comment 2：***the correlation structure presented here very likely depend on the context. This means, it depends what context is addressed (or referred to) in the items of the questionnaire, but also, what is the context where the sample population lives. The question is whether a general structure could be expected theoretically, apart from context differences, or whether different correlation structures are expected in different well-defined context. This point might be addressed a bit more.

**Response:** Thank you for pointing this out. We have added a discussion of this issue to the revised manuscript. Specifically, we discuss the generalisability and particularity of the correlation structures from three perspectives: different cultural contexts（p. 21, line 402-490）, different educational contexts and different sports contexts (p. 24, lines 491–510).

***Comment 3：***An interesting point would concern cultural differences in coaching styles. Typically, researchers distinguish between western and eastern cultures, and it would be interesting to know whether the concept of coaching style is viewed universal or specific with respect to culture.

**Response:** We do not develop culturally specific concepts of coaching style. However, using the original concept of the controlling coaching style, we examine whether Chinese and Western athletes have different responses to the controlling coaching style. We discuss the similarities and differences in the responses of Eastern and Western athletes towards the controlling coaching style on pages 21 to 22 (lines 02-490).

***Comment 4：***The hypotheses or research questions should be specified in more detail? What is expected on what theoretical grounds, and what are the ancillary variables that could affect the outcome?

**Response:** Thank you for this comment. We have added a discussion of the relevant assumptions to the revised manuscript (p. 4, lines 59–74).

***Comment 5：***More information should be provided regarding the used questionnaires. Do they mean the same after translation, for example, from english to chinese, or is this aspect examined in more detail?

**Response:** Thank you for this comment. We have added more information about the questionnaires in the revised manuscript (pp. 6–7, lines 113–122).

***Comment 6：***Statistics should be presented in tables and in a more systematic way. Measurements are taken on more than one occasion to study test-retest reliability of performance and subjective ratings? Before interpreting the correlation structure, I suggest considering Schumann's 7-point step guide as a general framework (doi: 10.3389/fpsyg.2022.946626, see Table 1). I suggest presenting a table with population parameters (M, SD, skew) of all variables, and the correlation in the MTMM style. It is important to know the test-retest reliability of the measures, also the reliability of the outcome solutions scales would be important to know. The authors are reporting cronbach alpha but it is important to have test-retest reliability on hand, but see my next point.

**Response:** We agree with this suggestion and have revised the tables accordingly (pp. 13–19). The study draws on Schumann’s 7-point guide as a general framework (Schumann et al., 2022). In Study 3, we examine the test-retest reliability of the scale (pp. 19–20).

***Comment 7：***reliability and cronbach alpha are different concepts. test-retest reliability refers to how precise the measurement is, so it means do we measure the same at different time points? reliability in terms of measurement precision is typically aimed to maximize, because high levels mean highest precision. This is different for cronbach alpha, which refers to the internal consistency of items under a scale. Here, high scores are not always wished because it would mean that a scale measure consists of very similar questions. therefore, cronbach's alpha is typically optimal when it matches the concept (small concept => high alpha, broadband concept = moderate alpha).

**Response:** Thank you for pointing this out. We have added a third study, Study 3, which measures the test-retest reliability of the Controlling Coach Behaviors Scale (pp. 19–20).

***Comment 8：***The discussion is a good read. Yet, the interpretation should be reconsidered a bit.

**Response:** Thank you very much for this suggestion. We have reorganised the discussion section to more clearly analyse the similarities and differences between Chinese and Western athletes’ perceptions of controlling coaching.

**Journal Requirements**

***Comment 1****:* *Please ensure that your manuscript meets PLOS ONE's style requirements, including those for file naming. The PLOS ONE style templates can be found at*
[*https://journals.plos.org/plosone/s/file?id=wjVg/PLOSOne_formatting_sample_main_body.pdf*](https://journals.plos.org/plosone/s/file?id=wjVg/PLOSOne_formatting_sample_main_body.pdf) *and*

[*https://journals.plos.org/plosone/s/file?id=ba62/PLOSOne_formatting_sample_title_authors_affiliations.pdf*](https://journals.plos.org/plosone/s/file?id=ba62/PLOSOne_formatting_sample_title_authors_affiliations.pdf)*"*

**Response:** Thank you for these links. We have modified the manuscript using the PLOS One style templates.

***Comment 2****:* *Please provide additional details regarding participant consent. In the ethics statement in the Methods and online submission information, please ensure that you have specified what type you obtained (for instance, written or verbal, and if verbal, how it was documented and witnessed). If your study included minors, state whether you obtained consent from parents or guardians. If the need for consent was waived by the ethics committee, please include this information.*

**Response:** We have added information about participant consent (p. 7, lines 125–126). All of the procedures performed in this study were approved by the Institutional Review Board of the Guangzhou Sport University. In all three studies, all of the participants or their parents signed informed consent forms.

***Comment 3****: PLOS requires an ORCID iD for the corresponding author in Editorial Manager on papers submitted after December 6th, 2016. Please ensure that you have an ORCID iD and that it is validated in Editorial Manager. To do this, go to ‘Update my Information’ (in the upper left-hand corner of the main menu), and click on the Fetch/Validate link next to the ORCID field. This will take you to the ORCID site and allow you to create a new iD or authenticate a pre-existing iD in Editorial Manager. Please see the following video for instructions on linking an ORCID iD to your Editorial Manager account:* [*https://www.youtube.com/watch?v=_xcclfuvtxQ*](https://www.youtube.com/watch?v=_xcclfuvtxQ)

**Response:** We have valid ORCID iDs, as required: https://orcid.org/0000-0002-2054-6911

***Comment 4****: Your ethics statement should only appear in the Methods section of your manuscript. If your ethics statement is written in any section besides the Methods,* ***Comment 4****please move it to the Methods section and delete it from any other section. Please ensure that your ethics statement is included in your manuscript, as the ethics statement entered into the online submission form will not be published alongside your manuscript.*

**Response:** Thank you for this information. We have moved the ethics statement to the Methods section (p. 7, lines 126–129).

***Comment 5****: We note you have included a table to which you do not refer in the text of your manuscript. Please* *ensure that you refer to Table 1 in your text; if accepted, production will need this reference to link the reader to the Table.*

**Response:** We have renamed the tables in this revision and have ensured that each one is referred to in the text (for example, p.3, lines 35).

***Comment 6****:* Please include a copy of Table 5 which you refer to in your text on page **Response:** We have renamed the tables in this revision, and we have ensured that each one is referred to in the text (for example, p.3, lines 35).

***Comment 7****: The //requires authors to make all data underlying the findings described in their manuscript fully available without restriction, with rare exception (please refer to the Data Availability Statement in the manuscript PDF file). The data should be provided as part of the manuscript or its supporting information, or deposited to a public repository. For example, in addition to summary statistics, the data points behind means, medians and variance measures should be available. If there are restrictions on publicly sharing data—e.g. participant privacy or use of data from a third party—those must be specified.*

**Response:** The data used in our experiments are available at https://osf.io/6rwts/. We have also added a table (Table 2-1 in the new version) that provides the means and standard deviations of the variables.
