# Supplementary material for: Examining the psychometric properties of the Controlling Coach Behaviors Scale in Chinese Elite Athletes
Source: PLoS One. 2022 Nov 28;17(11):e0277985. doi: 10.1371/journal.pone.0277985 (PMC9704685; doi:10.1371/journal.pone.0277985)
Supplement: Attachment — Submitted filename: Response to Reviewers.docx [file pone.0277985.s002.docx]

**Response to Reviewers**

We sincerely thank the editor and the reviewer for their comments and advice, which we have used to improve the manuscript. Below is a point-by-point response to each comment. The line numbers refer to the revised manuscript.

**Editor**

***Comment 1****:* As you can see from the review, R1 is satisfied and has only some very minor remarks. Based on my own reading, the manuscript is ready for publication and makes a good contribution to the field. I suggest preparing the final version of the manuscript and I will formally accept the paper in the next submission round. Please also check the reference list for completeness (e.g., doi number, page information, etc.). Also, Tables should be presented in APA style (e.g., headers, avoid vertical lines, etc.)

**Response:** Thank you very much for your affirmation of our manuscript. We have carefully checked the completeness of the reference list and corrected the writing style according to the requirements of the journal. No retracted papers are cited in the revised manuscript. In addition, we have reformatted the tables according to APA style guidelines.

**Reviewer**

***Comment 1****:* The manuscript has improved considerably and is in good shape and a good read overall. It is ready for publication and I have only a few very minor comments (see below).
168-169 "...the study draws on Schumann’s 7-point guide as a general framework (Schumann et al., 2022)...."At this occasion, I recommend adding a few more information, for example, in this way: The study draws on Schumann’s 7-point guide as a general framework (Schumann et al., 2022). Accordingly, molecular (skew check) and molar (reliability) preconditions are basically fulfilled. The questionnaires have been validated by previous research as sufficiently reliable (study) and also discriminates from other non-related concepts.

**Response:** Thank you very much for your support. We have added more information to the revised manuscript (pp. 10, 169–172).

***Comment 2****:* the discussion is very good! It is informative and insightful. Maybe the points could 1-7 could made more explicit. For example, at the occasion "-point 5- stability", it could be stated first what is the requirement in the ideal norm, and in the definition, and then discussing the issue further (what is the reality in the present case).

**Response:** Thank you for pointing this out. We have expanded the discussion on Points 1–7 (pp. 21, 401–422).
